# Use of the emergency department by refugees under the Interim Federal Health Program: A health records review

**DOI:** 10.1371/journal.pone.0197282

**Published:** 2018-05-10

**Authors:** Francis Bakewell, Sarah Addleman, Garth Dickinson, Venkatesh Thiruganasambandamoorthy

**Affiliations:** Department of Emergency Medicine, University of Ottawa, Ottawa, ON, Canada; Queensland University of Technology, AUSTRALIA

## Abstract

**Introduction:**

In June 2012, the federal government made cuts to the Interim Federal Health (IFH) Program that reduced or eliminated health insurance for refugee claimants in Canada. The purpose of this study was to examine the effect of the cuts on emergency department (ED) use among patients claiming IFH benefits.

**Methods:**

We conducted a health records review at two tertiary care EDs in Ottawa. We reviewed all ED visits where an IFH claim was made at triage, for 18 months before and 18 months after the changes to the program on June 30, 2012 (2011–2013). Claims made before and after the cuts were compared in terms of basic demographics, chief presenting complaints, acuity, diagnosis, presence of primary care, and financial status of the claim. Bivariate or multivariate logistic regression analysis was performed to yield odds ratios (OR) with 95% confidence intervals.

**Results:**

There were a total of 612 IFH claims made in the ED from 2011–2013. The demographic characteristics, acuity of presentation and discharge diagnoses were similar during both the before and after periods. Overall, 28.6% fewer claims were made under the IFH program after the cuts. Of the claims made, significantly more were rejected after the cuts than before (13.7% after vs. 3.9% before, adjusted OR 4.28, 95% CI: 2.18–8.40; p<0.05). The majority (75.0%) of rejected claims have not been paid by patients. Fewer patients after the cuts indicated that they had a family physician (20.4% after vs. 30% before, unadjusted OR 1.67, 95% CI: 1.14–2.44; p<0.05) yet a higher proportion of patients without a family physician were still advised to follow up with their family doctor during the after period (67.2% after vs. 41.8% before, unadjusted OR 2.85, 95% CI: 1.45–5.62; p<0.05).

**Conclusion:**

A higher proportion of both rejected and subsequently unpaid claims after the IFH cuts in June 2012, as demonstrated in the logistic regression analysis in this health records review, represents a potential barrier to emergency medical care, as well as a new financial burden to be shouldered by patients and hospitals. A reduction in IFH claims in the ED and a reduction in the number of patients with access to a family physician also suggests inadequate primary care for this population, yet this was not reflected in the follow-up advice offered by ED physicians to patients.

## Introduction

In 1957, Canada created the Interim Federal Health (IFH) Program, a medical insurance program for new immigrants, government-sponsored refugees (those whose refugee status was accepted prior to arrival in Canada), and refugee claimants [1]. Prior to 2012, this program provided financial coverage to all of these groups for medical care, vision care, emergency dental care, and prescriptions [2].

In June 2012, the Conservative federal government made cuts to the Interim Federal Health Program [3]. Refugee claimants would now only be covered for urgent or essential medical care, and only prescriptions for conditions considered a threat to public safety (e.g. tuberculosis) would be funded. A new subgroup of refugee claimants from ‘designated countries of origin’ (countries which the federal government deemed should not be producing refugees) [4], was also created, and would only receive coverage for medical care and prescriptions for conditions considered a threat to public safety [5].

The federal government defended the cuts as cost saving and as a deterrent for unfounded refugee claims [6]. The program cost $84.6 million in 2010–2011, and the cuts were projected to save $100 million over 5 years. Many professional groups protested the changes, claiming it would reduce access to care for an already marginalized population [7]. By early 2014, six provinces created their own temporary insurance programs to provide some medical coverage to refugee claimants [8]. In July 2014, the Federal Court of Appeal ruled that the cuts constituted “cruel and unusual” treatment, and were unconstitutional [9]. The federal government pledged to appeal the ruling, but by November 2014 it had reinstated some basic medical coverage for all groups [10]. On April 1^st^, 2016, the newly elected Liberal government fully restored both the eligibility and coverage to pre-2012 levels [11].

Several medical editorials at the time of the IFH cuts suggested that the impact on emergency departments (ED), may range from increased visits for non-emergency conditions, to late consequences of untreated chronic conditions [12–13]. This retrospective chart review sought to examine and describe the effect of the cuts on ED use among patients claiming IFH benefits, specifically looking at the nature and severity of chief presenting complaints, discharge diagnoses, rates of claim rejection, and existence of alternate means of primary care.

## Methods

### Design

We performed a health records review of all ED patients who had claimed IFH coverage at triage, comparing two 18 month periods, before and after the funding cuts came into effect on June 30^th^, 2012. Non-anonymized electronic medical records of ED visits were analyzed retrospectively. As a retrospective chart review, consent was excepted. The design was approved by the research ethics board.

### Setting

ED charts from the Ottawa Hospital (TOH) were reviewed. TOH is a multi-campus, adult tertiary care centre in Ottawa, Ontario, with an average annual ED census of approximately 150,000 visits.

### Population

Our study included all ED visits where a claim was made at triage for coverage under the IFH program, from January 1^st^, 2011 to December 31^st^, 2013. Approval for the study was granted by the Ottawa Hospital Research Ethics Board.

### Outcome measures

Data extracted from the electronic medical record of the ED chart included: basic demographics (age and sex); Canadian Triage and Acuity Score (CTAS); chief presenting complaint (standardized at triage); disposition (home vs. admission); discharge diagnosis; if there was a prescription on discharge; if family doctor follow-up was recommended; and if the patient had a family doctor documented on the ED chart.

Visit frequency for individual patients during the study period was also captured. The Canadian Triage and Acuity Score is a standardized triage scoring system adopted across Canada and in several other countries, wherein a score of 1 indicates the highest acuity patients who should be seen immediately, 2 indicates emergent patients who should be seen within 15 minutes, 3 indicates urgent patients who should be seen within 15 minutes, 4 indicates less urgent patients who should be seen within 60 minutes, and 5 indicates non-urgent patients who should be seen within 120 minutes [14]. Discharge diagnoses were recorded as written by the attending physician, and then grouped by system, acuity and severity for comparison.

The financial status of IFH claims for ED physician billings were reviewed. Rejected IFH claims were quantified and classified as paid or outstanding (referred to a collections agency). Hospital charges and payment status were also assessed for any claims that had been rejected by ED physician billing.

### Statistical analysis

Data was grouped for 18 months prior to the cuts (the ‘pre’ group) and 18 months after the cuts (the ‘post’ group). Statistical significance was determined using Fisher’s exact test, with significance noted at p<0.05. Bivariate or multivariate logistic regression analysis was performed to yield odds ratios (OR) with 95% confidence intervals.

## Results

### Demographics and frequency of ED use

IFH coverage was claimed for 612 patient visits to the TOH ED during the study period. There were 357 claims prior to the IFH cuts and 255 after (a 28.6% reduction). Accounting for repeat visits, there were 201 individual patients claiming IFH coverage before and 148 after (a 26.4% reduction).

Patient age, gender and visit frequency was similar between the two groups (Table 1).

**Table 1:** Demographics and frequency of ED use.

	PRE (%)	POST (%)
**Total IFH visits (n)**	357	255
**Repeat visits**	156 (43.7)	107 (42.0)
**Total IFH patients**	201	148
**Patients with multiple visits (> = 2)**	73/201 (36.3)	59/148 (39.9)
**Frequent users (> = 5/year)**	10/201 (5.0)	5/255 (2.0)
**Female**	202 (56.6)	134 (52.5)
**Median age (years)**	41	38
**Age >65**	36 (10.1)	22 (8.6)

### Diagnosis, severity and disposition

The presentations and diagnoses of patients were similar before and after the IFH cuts. Illness severity as assessed by the Canadian Triage and Acuity Scale (CTAS) and by hospital admission rates were similar in the two groups (Table 2). Prescriptions were given to patients at a similar rate before and after the cuts (39.7% and 46.3%, p = 0.136).

**Table 2:** Acuity of presentation and severity of diagnosis.

	PRE (%)	POST (%)
**CTAS Score 1**	0	0
**2**	82 (23.0)	61 (23.9)
**3**	169 (47.3)	123 (48.2)
**4**	81 (22.7)	57 (22.4)
**5**	19 (5.3)	7 (2.7)
Not recorded	6 (1.7)	7 (2.7)
**Disposition**		
**Home**	317 (88.8)	229 (89.8)
**Admission**	39 (10.9)	26 (10.2)
**Left Without Being Seen (LWBS)**	1 (0.3)	0 (0)

### Follow-up

After the cuts, only 20.4% of patients had access to a family physician (FP) documented on their ED chart, compared to 30.0% before (OR 1.67, 95% CI: 1.14–2.44; p = 0.009).

A higher, though statistically not significant proportion were advised to follow-up with their FP by the ED physician (26.3% vs. 22.1%, OR 1.25, 95% CI: 0.86–1.82), despite 67.2% of these patients not having an FP after the cuts, compared to 41.8% before (OR 2.85, 95% CI 1.45–5.62; p <0.003).

### Claim status

Claims to the IFH program for ED physician billings were rejected more frequently after the cuts (13.7%) than before (3.9%) (Table 3), with an unadjusted odds ratio of 3.9 (95% CI: 2.05–7.41, p<0.00001). After adjusting for age, sex, acuity, presence of FP, disposition, and CTAS score, the odds ratio is 4.28 (95% CI: 2.18–8.40), though none of these variables were significant. The multivariate logistic regression model is shown in Table 4.

**Table 3:** IFH claims status.

	PRE (%)	POST (%)	P value*	Unadjusted odds ratio (95% CI)	Adjusted odds ratio (95% CI)
**Total visits**	357	255			
**MD billings claims rejected**	14 (3.9)	35 (13.7)	0.00001	3.9 (2.05–7.41)	4.28 (2.18–8.40)
**Paid by patient**	7/14 (50.0)	7/35 (20.0)		4 (1.05–15.15)	
**Referred to collections agency**	7/14 (50.0)	7/35 (20.0)			
**Average cost of rejected claim**	$131.14	$115.76	N/A		
**Hospital costs claims rejected**	4 (1.1)	19 (7.5)	0.0006	7.1 (2.39–21.15)	8.31 (2.69–25.66)
**Paid by patient**	1/4 (25.0)	3/19 (15.8)		1.78 (0.14–23.42)	
**Referred to collections agency**	3/4 (75.0)	16/19 (84.2)			
**Average cost of rejected claim**	$990.04	$726.88	N/A		
**Total cost of rejected claims**	$5,796.10	$17,862.51	N/A		

*Only significant p values included

**Table 4:** Multivariate analysis for MD billings claims–rejected.

	β coefficient	p-value	Odds ratio (95% CI)
Intercept	-2.49	0.01	—
Pre/Post Cuts Cohort	1.45	< .0001	4.28 (2.18–8.40)
Age	0.00	0.87	1.00 (0.98–1.02)
Sex	-0.44	0.16	0.64 (0.35–1.19)
Acuity	-0.33	0.29	0.72 (0.39–1.32)
Presence of FP	0.46	0.19	1.59 (0.80–3.15)
Disposition	1.10	0.04	3.02 (1.04–8.79)
CTAS	-0.14	0.51	0.87 (0.57–1.33)

Fewer of these rejected claims had been paid by patients after the cuts (20.0%) compared to before (50.0%) (OR 4, 95% CI: 1.05–15.15), however this did not reach statistical significance (p = 0.076).

Similarly, of the claims made to the IFH program for hospital costs, more were rejected after the cuts (7.5%) than before (1.1%), with an unadjusted odds ratio of 7.10 (95% CI: 2.39–21.15; p <0.0006). After adjusting for age, sex, acuity, presence of FP, disposition, and CTAS score, the odds ratio is 8.31 (95% CI: 2.69–25.66), with only the presence of an FP reaching significance. The multivariate logistic regression model is shown in Table 5.

**Table 5:** Multivariate analysis for hospital costs claims–rejected.

	β coefficient	p-value	Odds ratio (95% CI)
Intercept	-4.31	0.003	—
Pre/Post Cuts Cohort	2.12	0.0002	8.31 (2.69–25.66)
Age	0.00	0.86	1.00 (0.97–1.03)
Sex	-0.54	0.23	0.58 (0.24–1.42)
Acuity	-0.54	0.21	0.58 (0.25–1.35)
Presence of FP	1.33	0.004	3.77 (1.52–9.37)
Disposition	-0.38	0.75	0.68 (0.07–6.75)
CTAS	0.05	0.87	1.05 (0.58–1.89)

Again, fewer of these rejected claims had been paid by patients after the cuts (15.8%) compared to before (25.0%) (OR 1.78, 95% CI: 0.14–23.42), however this did not reach statistical significance.

The total of rejected claims was $5,796.10 before the cuts and $17,862.51 after the cuts.

It is unclear why there was a discrepancy between the number of MD billings claims that were rejected by the IFH program compared to the number of hospital cost claims rejected. The claims are submitted by two separate offices and processes in the hospital, and would have been reviewed separately by the IFH program.

## Discussion

The introduction of restrictions in eligibility for the IFH program in June 2012 resulted in a decline in total claims for ED visits, and an increase in rejected claims, both for ED physician billings and hospital costs. This may be a reflection of patient uncertainty as to whether or not they were still eligible for emergency medical care following the IFH cuts.

The changes to the IFH program did not affect the basic demographics of patients accessing the ED, nor their diagnoses, acuity, severity of illness or disposition.

After the cuts, only 20.4% of patients indicated that they had a family physician. This was a 32% relative decrease from before the cuts and may have been a consequence of the reduction in IFH eligibility for primary care.

Despite lacking documentation of a primary care provider on the emergency record, many patients were still advised to follow-up with one. This suggests a need to provide better emergency department discharge advice to this group of patients.

This study describes the impact of the 2012 cuts to the IFH program on ED use by adult refugee claimants. Evans et al. published a similar study in 2014 on the impact of the cuts on claims made in the pediatric ED at the Hospital for Sick Children in Toronto [15]. Similar to our study, they found that the IFH program cuts, reduced the number of claims and increased rejected claims. They also included the costs of hospital admission, which resulted in a much greater financial impact than ED costs alone. Likewise, a study by Bozorgmehr and Razum of the health expenditures of asylum seekers and refugees between 1994 and 2013 showed that those with restricted access to healthcare benefits from the state ultimately incurred greater overall costs than those who had unrestricted access from the start [16].

A significant limitation of our study is that it reflects only those patients who made claims under the IFH program at triage, rather than all refugees seeking care. It is therefore unknown if refugee claimants who had their IFH coverage revoked still visited the ED as ‘self-pay’ patients, or if they avoided emergency care completely due to an inability to pay. Similarly, some of the patients who had their claims rejected may not have been ineligible due to the changes in coverage, but may have had their refugee application denied. Another limitation to the study is that older rejected claims (including those rejected before the cuts) have had more time to be paid by patients, and so differences in payment status may simply reflect the passage of time rather than ability to pay.

One of the stated goals of the cuts to the IFH program was to save the federal government money [17]. By that metric, the near 30% reduction in the number of claims and the increase in the number of rejected claims following the cuts could be considered a success. However, while the absolute value of unpaid claims ($17,862.51 after the cuts) may not seem significant when considered in the context of hospital budgets, the average amount billed for each visit ($115.76 for MD billings and $726.88 for hospital costs after the cuts) may indeed be significant for a refugee patient with little or no income. This is supported by the proportion of rejected claims (e.g. 80% of claims for MD billings) that remained unpaid by patients. The savings to the federal government were passed on to the refugee claimant patients, the emergency physicians providing care and the hospital/provincial government [18]. The concept of cost shifting is well recognized in American healthcare, where administrative costs can be downloaded on to patients and their insurers, but is seen less often in single payer systems [19]. This would seem to be a unique Canadian example, however, despite being originally described as cost saving by the federal government [20].

While the 2012 changes to the Interim Federal Health Program have been reversed, this study describes the impact that cuts to the health insurance program had on access and use of the emergency department by refugee claimants and the cost consequences to patients, emergency physicians and hospitals.

## Supporting information

S1 Dataset Use of the emergency department by refugees under the Interim Federal Health Program dataset. (XLSX)
